# Characterizing the landscape of cervical squamous cell carcinoma immune microenvironment by integrating the single‐cell transcriptomics and RNA‐Seq

**DOI:** 10.1002/iid3.608

**Published:** 2022-05-11

**Authors:** Ruiling Yin, Xiuming Zhai, Hongyan Han, Xuedong Tong, Yan Li, Kun Deng

**Affiliations:** ^1^ Department of Laboratory Medicine The Third Affiliated Hospital of Chongqing Medical University Chongqing China

**Keywords:** cervical cancer, immunotherapy, single‐cell sequencing, TAM

## Abstract

**Background:**

Cervical squamous cell carcinoma (CSCC), caused by the infection of high‐risk human papillomavirus, is one of the most common malignancies in women worldwide.

**Methods:**

RNA expression data, including those from the Cancer Genome Atlas, Gene Expression Omnibus, and Genotype‐Tissue Expression databases, were used to identify the expression of RNAs in normal and tumor tissue. Correlation analysis was performed to identify the immune‐related long noncoding RNAs (IRLs) and hypoxia‐related genes (IRHs) that can influence the activity of the immune system. Prognosis models of immune‐related RNAs (IRRs) were used to construct a coexpression network of the immune system. We identified the role of IRRs in immunotherapy by correlation analysis with immune checkpoint genes (ICGs). We then validated the expression data by integrating two single‐cell sequencing data sets of CSCC to identify the key immune features.

**Results:**

In total, six immune‐related gene (IRG), four IRL, and five IRH signatures that can significantly influence the characteristics of the tumor immune microenvironment (TIME) were selected using machine learning methods. The expression level of ICGs was significantly upregulated in GZMB^+^CD8^+^ T‐cells and tumor‐associated macrophages (TAMs) in tumor tissues. TGFBI^+^ TAMs are a kind of blood‐derived monocyte‐derived M0‐like TAM linked to hypoxia and a poor prognosis. IFI30^+^ M1‐like TAMs participate in the process of immune‐regulation and showed a role in the promotion of CD8^+^ T‐cells and Type 1 T helper (Th1)/Th2 cells in the coexpression network, together with several IRLs, IRGs, and ICGs.

**Conclusions:**

CD16^+^ monocyte‐derived IFI30^+^ TAMs participated in our coexpression network to regulate the TIME, showing the potential to be a novel immunotherapy target. The enrichment of M0‐like TAMs was associated with a worse prognosis in the high‐risk score group with IRH signatures. Remarkably, M0‐like TAMs in tumor tissues overexpressed *TGFBI* and were associated with several well‐known tumor‐proliferation pathways.

## Graphical abstract

Cervical squamous cells carcinoma (CESC), caused by the infection of high‐risk human papillomavirus (HR‐HPV). We investigate a combination of multiple immune biomarkers as a clinically signature that can predict prognosis, diagnosis, relapse, and therapy in CESC patients.

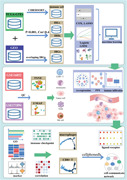

## 1 INTRODUCTION

Cervical cancer (CC) is one of the common types of gynecological malignancies, ranking second only to breast cancer.^1^ The incidence and mortality rates of CC have increased year by year and more and more younger individuals are being diagnosed in urban China.^2^ Viral infection induces various immune responses in the host to control viral replication, but it does not necessarily lead to CC.^3^

Tumors are complex ecosystems defined by spatiotemporal interactions between different cell types.^4^ The tumor microenvironment (TME), which consists of vascular vessels, fibroblasts, endothelial cells, distinct infiltrating immune cell (IIC) subsets,^5^ bone marrow‐derived progenitors, platelets, and inflammatory cytokines,^6^, ^7^ plays a vital role in the development of tumor tissues.^8^, ^9^, ^10^ The tumor immune microenvironment (TIME) and certain IIC types could lead to a poor prognosis.^11^, ^12^ Immune checkpoint inhibitors (ICIs) are one of the most effective anticancer treatment methods available today.^13^, ^14^, ^15^ However, the composition of the TIME can directly reflect treatment efficacy.^16^ Proinflammatory cytokines secreted by tumor cells and IICs have been shown to regulate tumor progression and immune evasion.^17^ Hypoxia strongly stimulates the TME of CC,^18^ inducing the trafficking of macrophages—especially M2‐like phenotype macrophages^19^, ^20^—into tumor areas^21^ and contributing to cell–cell communication,^22^ thereby inducing immunosuppression.^23^ Long noncoding RNAs (lncRNAs) of >200 nucleotides^24^ play a key role in the pathogenesis of several kinds of cancers.^25^, ^26^, ^28^ Despite growing appreciation of the importance of lncRNAs in disease,^26^ our knowledge of the contact between immune‐related lncRNAs (IRLs) and the TIME in CC remains limited. The Cancer Genome Atlas (TCGA) and Genotype‐Tissue Expression (GTEx)^29^ which generated matching normal tissue data for various human tissues,^30^ provide a wide range of data mining capabilities for gene functions.

In recent years, machine learning (ML) has become widely used in various fields.^31^, ^32^, ^33^, ^34^ We sought to investigate the roles of TIME in the development of CC, adopting the use of a ML method for screening the prognostic significance of some immune‐related RNAs (IRRs) and IICs. Due to the TIME hypoxia circumstances, we analyzed the correlation between hypoxia‐related genes (HRGs) and immune‐related genes (IRGs). We observed decreased levels of CD8^+^ T‐cells and increased fractions of M0‐like macrophages in the high‐risk score group of immune‐related hypoxia‐related gene (IRH) signatures compared to those in the low‐risk score group. The coexpression network was constructed by IRRs. Single‐cell analysis has revealed the heterogeneity of immune cells in a variety of cancer types.^35^, ^36^, ^37^, ^38^ We identified the expression of these genes and existing immunotherapy sites in different states of cells by pseudotime using single‐cell RNA sequencing (RNA‐seq). CellPhoneDB revealed the importance of tumor‐associated macrophages (TAMs) in TIME cell communications. We investigated a combination of multiple immune biomarkers as a clinically relevant signature that may predict prognosis, diagnosis, relapse, and therapy in CC patients.

## 2 MATERIALS AND METHODS

### 2.1 Data acquisition

TCGA and GTEx count data, TCGA–cervical squamous cells carcinoma (CESC) fragments per kilobase of transcript per million mapped reads (FPKM) data, and corresponding clinical information were downloaded from the Xena database (https://xenabrowser.net/) of the University of California. The TCGA–CESC FPKM sequence data were translated to TPM sequence data. Meanwhile, RNA‐seq microarray datasets (GSE138080 and GSE63514) and clinical information of cervical squamous cell carcinoma (CSCC) were downloaded from Gene Expression Omnibus (GEO) (https://www.ncbi.nlm.nih.gov/gds). GSE138080 obtained 10 healthy cervical tissues and 10 CSCC. GSE63514 obtained 24 healthy cervical tissues and 28 tumor tissues (27 CSCC and 1 missing). GSE168652 includes two samples (CSCC tissues and adjacent normal tissues). GSE171894 contains four fresh samples from patients with CSCC. Also, Ensembl identifiers were translated to gene symbols by annotation files (gencode.v22.annotation.gtf3.gz) downloaded from Gencode (https://www.gencodegene.org/) to distinguish between the messenger RNA (mRNA), microRNA, and lncRNA.

### 2.2 Estimation of TME signatures

CIBERSORT is a gene‐based deconvolution algorithm^39^ that can infer 22 human immune cell types. The TPM expression datasets were uploaded to R software, and we used the characteristics of 547 marker genes and 1000 permutations to quantify the proportion of each cell type.

### 2.3 Identification of IRGs in TCGA–GTEx and GEO datasets

The IRGs were downloaded from the IMMPORT database (https://www.IMMPORT.org/). The common Cuffdiff analysis packages (DESeq. 2) were used to identify the differentially expressed genes (DEGs) (|LogFC| > 1, false discovery rate < 0.05) in the TCGA–GTEx cohort. The GEO chip microarray data (GSE138080 and GSE63514) were analyzed using the R limma package.

### 2.4 Coexpression to define the immune‐related lncRNAs and hypoxia genes

A coexpression strategy (*p* < .001, *F* > 0.4) was applied to confirm the correlation efficiency in screening the IRLs and IRHs in TPM sequence data. A total of 200 HRGs were downloaded from MSigdb hallmark gene sets (http://www.gsea-msigdb.org/gsea/msigdb/).

### 2.5 Establishment and validation of IRG signatures

We used traditional ways to construct IRG prognosis models to predict the prognosis of cervical patients. Those patients with full survival information were randomly divided into training and testing sets. Same as in the above method, the IRGs were selected by univariate Cox regression and Lasso regression. The risk score models were constructed by multivariable Cox regression. The TCGA cohort was stratified into high‐risk and low‐risk groups based on the median risk score. The difference in overall survival (OS) between the two groups was calculated by the Kaplan–Meier method. The receiver operating characteristic (ROC) curve was constructed using the survival ROC package in R to analyze the predictive accuracy. The prediction of it was analyzed by the Kaplan–Meier logrank test and time‐dependent ROC curve analysis. Hazard ratios (HRs) <1 indicated protective genes, while HRs of <1 indicated risk genes. Then, the risk models were built based on multivariable Cox models. The formula of risk score calculation was as follows:

Riskscore=∑βigenei×expression(genei).

### 2.6 Analysis of the correlation between immune checkpoint gene expression and immune profiles

Immune checkpoint genes (ICGs) associated with IRRs were downloaded from the TISIDB database (http://cis.hku.hk/TISIDB/) with the goal of analyzing the interactions between tumors and the immune system. Immunoinhibitors and immunostimulators that were significantly correlated with IRRs in terms of gene expression were selected (*p* < .05).

### 2.7 Quality control of single‐cell sequencing data and cell types defined

GSE168652 includes two samples (CSCC tissues and adjacent normal tissues) from a 53‐year‐old patient with human papillomavirus 18‐positive CSCC. GSE171894 contains four fresh samples from patients with CSCC who underwent concurrent chemoradiotherapy.

The top 2000 variable genes were used in the follow‐up analysis. Cell markers were downloaded from cellsMarker (https://www.labome.com/method/cells-Markers.html) and existing literature to distinguish different cell types. The R package Seurat was used to complete the initial quality control process (i.e., to screen for a gene expressed in ≥3 cells, mitochondrial gene expression <10%, and select cells with ≥200 genes).

### 2.8 Pseudotime trajectory analysis

The Monocle2 package (version 2.8.0) was used to analyze single‐cell trajectories to discover the cell state transitions. The trajectory was visualized as a two‐dimensional t‐distributed stochastic neighbor embedding (t‐SNE) graph, and dynamically expressed heatmaps were constructed using the plot_pseudo‐time_heatmap function.

### 2.9 Cell–Cell interaction analysis

CellPhoneDB^40^, ^41^ is a Python‐based computational analysis tool; it can be used to analyze cell–cell communications between cells at the molecular level. Considering the cell types or clusters, CellPhoneDB was used to analyze the major cell types and cell subclusters in normal tissues and TME.

### 2.10 Protein–protein interaction network

A protein–protein interaction (PPI) network was created using the STRING database (https://string-db.org/). The output data were entered into Cytoscape (version 3.8.2) to construct the PPI network.

### 2.11 Statistical analysis

Data were analyzed by R (version 3.6.3). The Venn plot was drawn by Venny (https://bioinfogp.cnb.csic.es/tools/venny).

### 2.12 mRNA and protein expression and immunochemistry analysis

The protein expression immunochemistry image was obtained from online datasets The Human Protein Atlas (THPA, https://www.proteinatlas.org/). CancerSEA is a database designed to decode 14 functional states of cancer cells at a single‐cell resolution, which includes 41,900 cancer single cells from 25 cancer types.^42^

## 3 RESULTS

### 3.1 Clinic information and the study process flow

**Figure 1:**
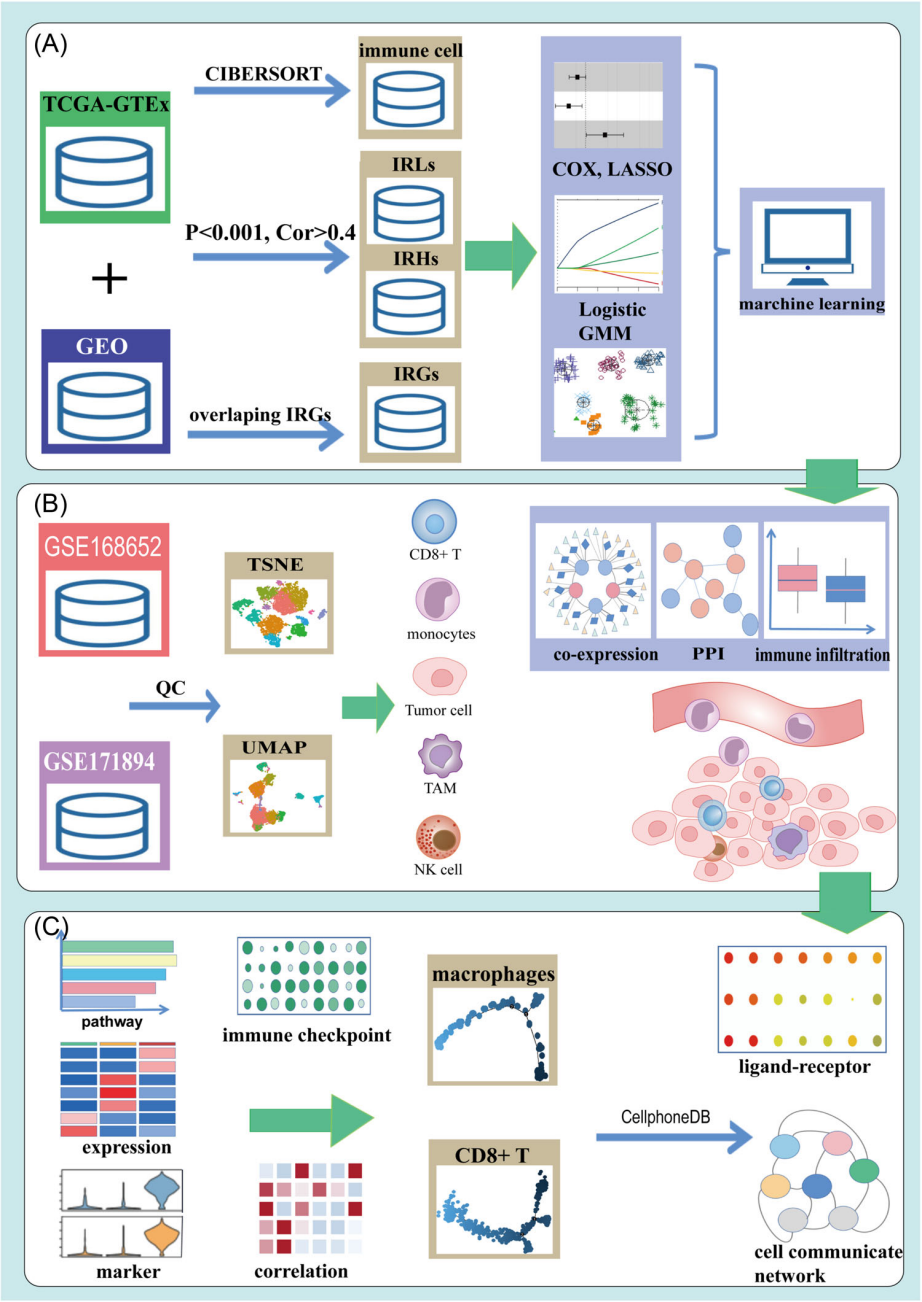
The workflow of this study. (A) Screening of immune‐related RNAs (IRRs) and immune cells with prognostic value. (B) Single‐cell sequencing to identify the location of the IRRs. (C) IRRs between cell–cell communications. GEO, Gene Expression Omnibus; GMM, Gaussian mixture model; GO, Gene Ontology; GTEx, Genotype‐Tissue Expression; IRG, immune‐related gene; IRH, immune‐related hypoxia‐related gene; IRL, immune‐related long non‐coding RNA; NK, natural killer; PPI, protein–protein interaction; TAM, tumor‐associated macrophage; TCGA, The Cancer Genome Atlas; TSNE, t‐distributed stochastic neighbor embedding; UMAP, uniform manifold approximation and projection

The workflow of this study is shown in Figure 1. The transcriptome profiling data of CC included datasets from the TCGA database, which contains 306 tumor data and 3 adjacent normal tissue data, and 10 normal tissues from the GTEx database. Chip microarray datasets (GSE138080 and GSE63514) were downloaded from the GEO database. The CIBERSORT deconvolution algorithm was used to quantify the fraction and infiltration of 22 kinds of immune cells in CC. Machine learning (ML) methods were used for the screening of IRGs with prognostic value (Figure 1A). We also used Cytoscape and R packages to analyze the coexpression network and gene enrichment pathway to trace down genes related to inflammation and immune‐regulation. Single‐cell sequencing raw data (GSE16852 and GSE171894) were downloaded from GEO. Then, we manually divided the cell clusters according to different cell types to identify the expression of IRRs in the TME (Figure 1B) and quantify the difference between normal and tumor tissues.

In Figure 1C, the pseudotime trajectory analysis was applied to CD8^+^ T‐cells and macrophages in two single‐cell sequencing datasets to analyze the genetic changes of CD8^+^ T‐cells and macrophages in normal and tumor tissues and at different stages of tumor development. CellPhoneDB was used to calculate the differences in ligand–receptors between different cells in the TME. Then, we calculated the correlations between ICGs and immune cells. In this study, we aimed to discern the differences in IRR expression between the TCGA–GTEx and GEO databases to determine the potential prognostic value of the DEGs between tumor and normal tissues.

### 3.2 Identification of IRGs in CC

**Figure 2:**
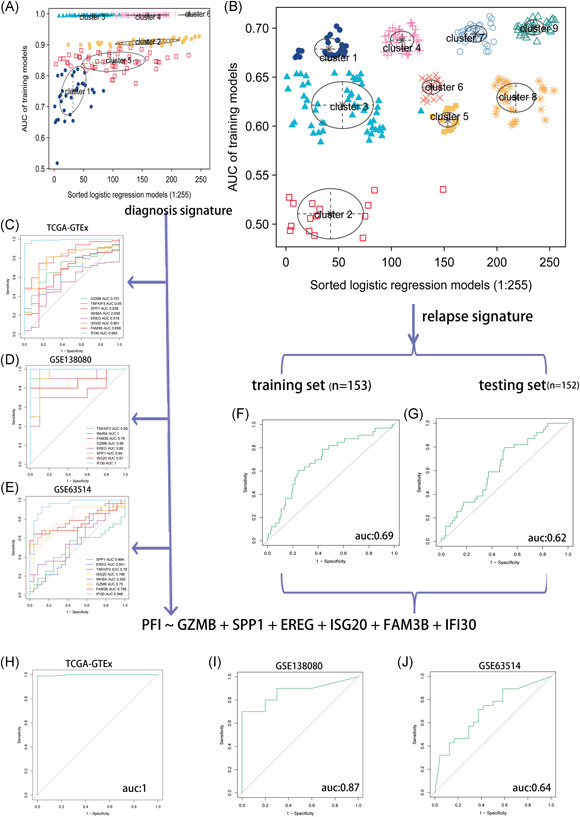
Receiver operating characteristic (ROC) curve plot of each Gaussian regression to distinguish the tumor and normal tissues. (A) The logistic regression of immune‐related gene (IRG) diagnosis models in the Cancer Genome Atlas (TCGA) cohort. (B) The logistic regression of IRG collapse models in the TCGA cohort. (C–E) ROC curve plot of each immune gene to distinguish tumor and normal tissues. TCGA–Genotype‐Tissue expression (GTEx), GSE138080, and GSE62514. (F,G) ROC curve plot showing the predictive value of diagnosis models for cervical squamous cell carcinoma recurrence. (H–J) ROC curve plot showing the diagnosis models to distinguish the tumor from normal tissues and predict the relapse. AUC, area under the ROC curve

There were 36 overlappings differentially expressed IRGs between normal and tumor tissues in three cohorts (TCGA–GTEx, GSE138080, and GSE63514). First, 11 DEIs associated with OS were selected by univariate Cox regression (*p* < .05). Second, we removed DEIs (*p* > .05) that showed no significant effect on prognosis in public online databases (Gene Expression Profiling Interactive Analysis [GEPIA]). Finally, we built a diagnosis signature by logistic regression based on eight DEIs, and area under the ROC curve (AUC) values (best models) were selected by a Gaussian mixture model (GMM‐based clustering). There were a total of 255 different gene‐combination formulas and a total of 255 logistic regression models (Figure 2A,B). We calculated the best diagnosis models by GMM with the R mclust package (Figure 2A) in the TCGA–GTEx cohort, and the AUC (*T* = 305, *N* = 13) was 1 (Figure 2H). We predicted the model in the validation sets (GSE138080 and GSE63514), and the AUCs were 0.87 and 0.64 (Figure 2I,J). We used the same six genes (Figure 2C–E) to build a logistic regression model to predict the relapse rate (Figure 2B). A total of 292 patients were divided into a training set (*n* = 153) and a testing set (*n* = 152) randomly; then, we calculated the Gaussian distribution of the relapse rates of the two sets and found that, in Cluster 9 of the training set, the prediction rate was 0.69, while the prediction rate of the testing set was 0.62 (Figure 2F,G). Through the construction of the diagnostic model and the construction of the prediction model, we found that there were six IRGs with good rates in predicting recurrence and distinguishing normal tissues from tumor tissues. Logistic regression models were calculated according to the combination of the following genes to predict the relapse: *GZMB* + *SPP1* + *EREG* + *ISG20* + *FAM3B* + *IFI30*. These six IRG signatures can also predict diagnosis and prognosis.

### 3.3 IRH prognosis models in the TCGA cohort

**Figure 3:**
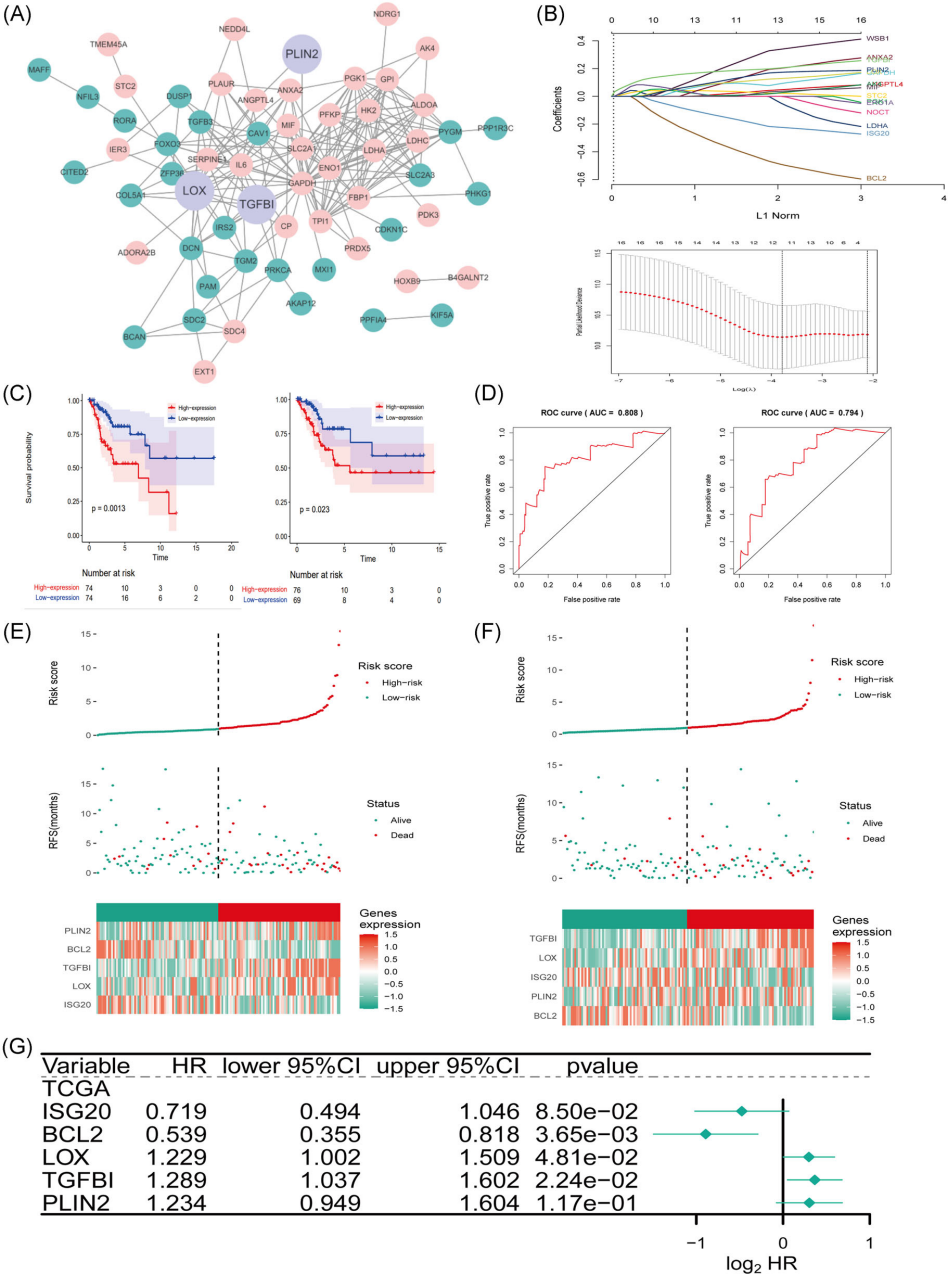
Immune‐related hypoxia gene signatures in The Cancer Genome Atlas (TCGA) cohort. (A) STRING‐based protein–protein interaction network of proteins of immune‐related hypoxia‐related genes (IRHs). (B) Cross‐validation in the Lasso analysis; Lasso coefficient profiles of the IRHs in the training set. (C) Kaplan–Meier analysis (survival plot) for risk groups. (D) The 1‐year time–receiver operating characteristic (ROC) curve plot of hypoxia‐related signatures. (E) Distribution, survival status, and heatmap of high‐ and low‐risk patient groups in the training set. (F) Distribution, survival status, and heatmap of high‐ and low‐risk patient groups in the testing set. (G) Forest plot of IRH signatures in the training set. AUC, area under the ROC curve; CI, confidence interval; HR, hazard ratio; RFS, recurrence‐free survival

Pearson's analysis (corFilter = 0.4, *p* < .001) revealed that most (65/76 DEGs) of the HRGs had a very strong association with 597 differentially expressed IRGs in TCGA corhorts. These IRHs include 32 downregulated genes and 44 upregulated genes. With the univariate Cox regression and Lasso algorithm (Figure 3B), we finally established an IRH signature risk model by finding five IRH signatures to predict the prognosis of CC. The risk score formula was as follows: [*BCL2* expression × (−0.6179)] + [*ISG20* expression × (−0.3295)] + [*TGFBI* expression × (0.2535)] + [*PLIN2* expression × (0.2101)] + [*LOX* expression × (0.2065)]. Patients in the training set and testing set were divided into two high‐ and low‐risk groups according to the median risk score. Kaplan–Meier survival analysis (Figure 3C) indicated that patients in the high‐risk group had the worst survival in the training (*p* = .0013) and testing (*p* = .023) sets. The AUC values were 0.808 and 0.794, respectively, for the training set and testing set (Figure 3D). The significance of the risk score was proved in the multivariate Cox analysis (Figure 3G). *BCL2* and *ISG20* were low‐risk immune genes in CC, while *TGFBI*, *PLIN2*, and *LOX* were high‐risk immune genes. Among these genes, *TGFBI*, *PLIN2*, and *LOX* showed interactions with IRHs (Figure 3A).

### 3.4 Constructed coexpression network

**Figure 4:**
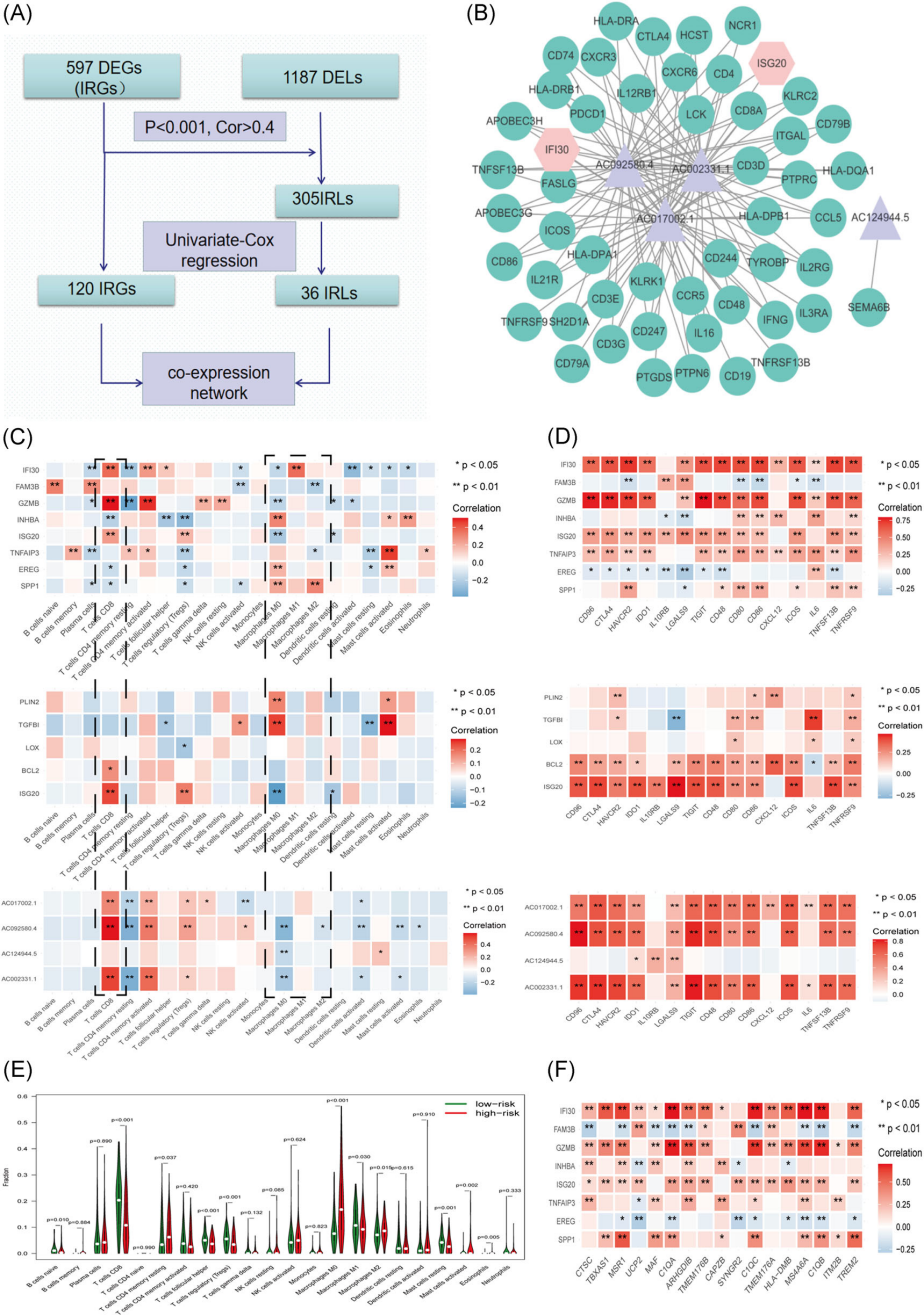
(A) The workflow of the coexpression network. (B) Coexpression network between immune‐related long noncoding RNAs (IRLs) and immune‐related gene (IRGs). (C) Correlations between the immune‐related RNAs and infiltrating immune cell subtypes: IRGs, immune‐related hypoxia‐related genes, and IRLs. (D) The Kaplan–Meier survival plots of *SPP1*, *IFI30*, and *TGFBI*. (E) Comparisons of immune cells between high‐ and low‐risk score groups. (F) The boxplot showing that the correlations between the eight IRGs and CIQC‐TAM signatures. DEG, differentially expressed gene; DEL, differentially expressed lncRNAs; TAM, tumor‐associated macrophage

Following univariate Cox regression analysis, 4 prognosis‐related IRLs and 51 prognosis‐related IRGs were selected and analyzed to construct the coexpression network (Figure 4A,B). It could be seen that hub lncRNAs (AC002331.1, AC017002.1, AC092580.4, and AC124944.5) and IRGs (*IFI30* and *ISG20*) were positively correlated with a variety of ICGs (*CTLA4*, *TNFRSF9*, *CD86*, *TNFRSF13B*, *PDCD1*, and *CD48*) and marker genes of different immune cells, such as CD8^+^ T‐cells (*CD8A*, *CD3D*, *CD79A*, and *CD3E*) and B‐cells (*CD19*), forming a network of connections between myeloid cells and T‐cells. This indicates that the complexity of IFI30^+^ TAMs in promoting immune effects, which may cause T‐cells to fatigue while stimulating T‐cell toxicity. A boxplot (Figure 4C) shows the correlation between multiple IRRs and immune infiltration cells; the blue box indicates a negative correlation, whereas the red box indicate a positive correlation. *ISG20*, *IFI30*, *GZMB*, AC092580.4, AC017002.1, AC002331.1, and CD8^+^ T‐cells are positively correlated. *SPP1* is positively correlated (*p* < .01) with M2‐like macrophages. *IFI30* is positively correlated (*p* < .01) with M1‐like macrophages. *TGFBI* is positively correlated (*p* < .01) with M0‐like macrophages. For IRHs (Figure 4E), CIBERSORT analysis results showed that significantly higher M0‐like macrophage cell counts (*p* < .001) appeared in the high‐risk score group relative to the whole cohort, with a lower percentage of CD8^+^ T‐cells (*p* < .001). A total of 14,852 lncRNA expression profiles in the TCGA–GTEx dataset were obtained in this study. A total of 305 IRLs were screened out by coexpression analysis from 1187 differentially expressed lncRNAs. The correlations between IRGs and ICG are shown in Figure 4C, indicating that *IFI30*, *GZMB*, *ISG20*, AC092580.4, AC017002.1, and AC002331.1 are strongly correlated with ICGs (most of them *p* < .01, cor > .7). The correlations (Figure 4F) between the eight IRG signatures and CIQC‐TAM signatures are shown in Figure 4F, indicating that *IFI30* and *GZMB* are significantly positively correlated with CIQC‐TAMs and many IRGs, while *SPP1* had no significant correlation with IRGs.

### 3.5 The relationship between IRRs and immune cells

**Figure 5:**
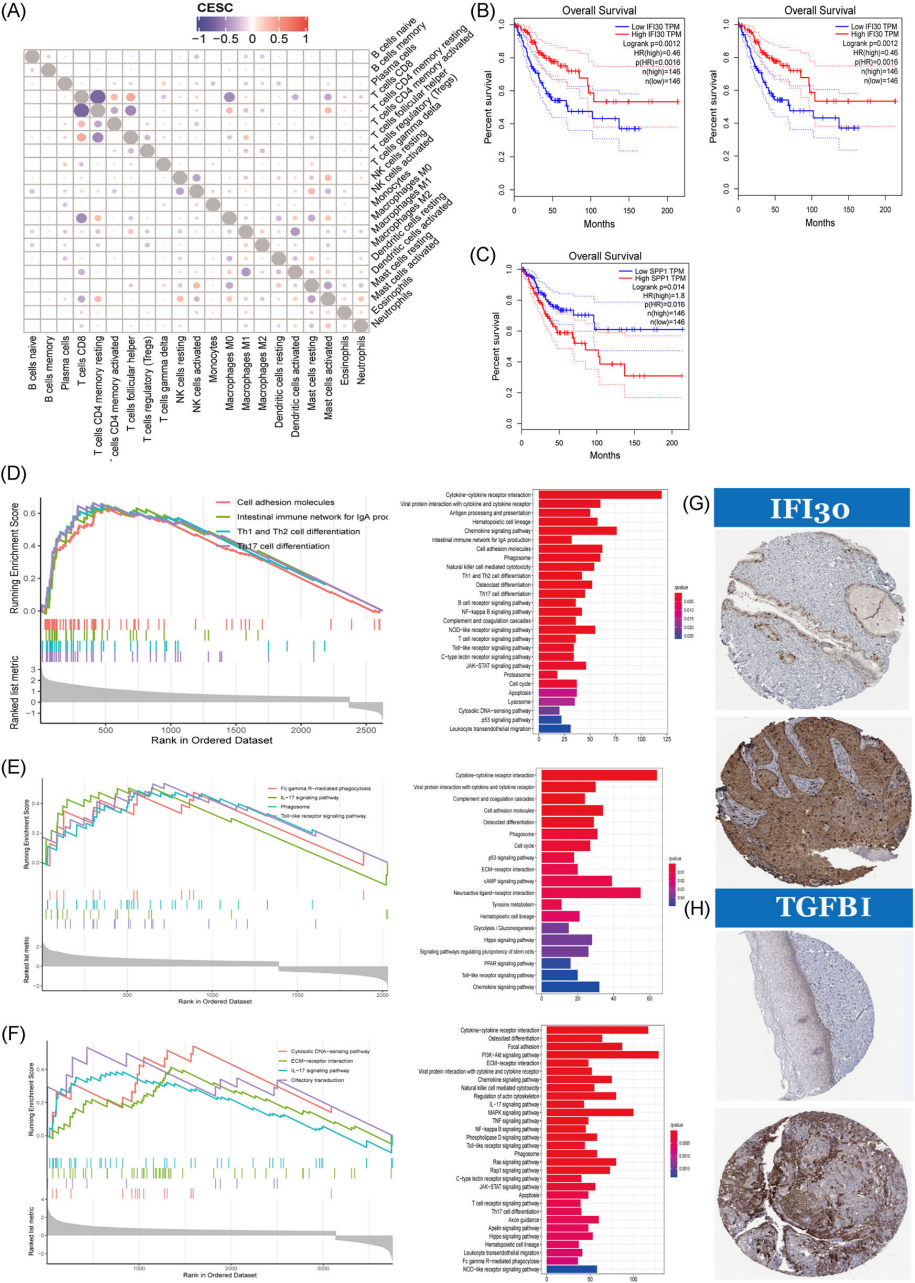
Characteristic of immune‐related RNAs in tumor‐associated macrophages. (A) Correlations between different infiltrating immune cell subtypes. (B,C) The Kaplan–Meier survival plots of *IFI30*, *TGFBI*, and *SPP1* (Gene Expression Profiling Interactive Analysis). (D) The gene set enrichment analysis (GSEA) plot of *IFI30*. (E) The GSEA plot of *SPP1*. (F) The GSEA plot of *TGFBI*. (G) Immunohistochemical map of *IFI30* protein. (H) Immunohistochemical map of *TGFBI* protein. CESC, cervical squamous cells carcinoma; ECM, extracellular matrix; HR, hazard ratio; IgA, immunoglobulin A; IL‐17, interleukin 17; JAK, Janus kinase; MAPK, mitogen‐activated protein kinase; NF‐κB, nuclear factor‐κB; NK, natural killer; NOD, nucleotide oligomerization domain; PPAR, peroxisome proliferator‐activated receptor; STAT, signal transducer and activator of transcription; Th1, Type 1 T helper; TNF, tumour necrosis factor

The corplot (Figure 5A) indicated that CD8^+^ T‐cells are negatively correlated with CD4^+^ memory T‐cells and M0‐like TAMs. The high expression levels of *TGFBI* and *SPP1* are related to a poor prognosis, while the high expression level of *IFI30* is related to a good prognosis via the GEPIA database (Figure 5B,C). The GSEA plots of *IFI30*, *SPP1*, and *TGFBI* are shown in Figure 5D–F. The immune process‐related pathway was significantly enriched in subgroups divided by the midexpression of *IFI30*. In particular, pathways that promote the differentiation of Th1, Th2, B‐cells, and cells of a hematopoietic lineage are enriched in the high‐level group of *IFI30*. Meanwhile, in subgroups divided by the expression level of *SPP1*, tumor proliferation‐related pathways, such as P53 signaling pathways or cell cycle pathways, were enriched in high‐expressed level groups. In subgroups divided by the expression levels of *TGFBI*, tumor immunosuppression‐related pathways, such as P53 signaling pathways, phosphoinositide 3‐kinase–protein kinase B (PI3K–AKT) signaling pathways, cell cycle pathways, tumor necrosis factor (TNF)‐κB signaling pathways, Janus kinase–signal transducer and activator of transcription signaling pathways, and apoptosis pathways, were enriched in high‐level expression groups. The expressions of *IFI30* and *TGFBI* in tumor and normal tissues were verified by THPA database (Figure 5G,H).

### 3.6 Single‐cell sequencing to define the expressions of IRRs and ICGs of immune‐infiltrating cells in TME

**Figure 6:**
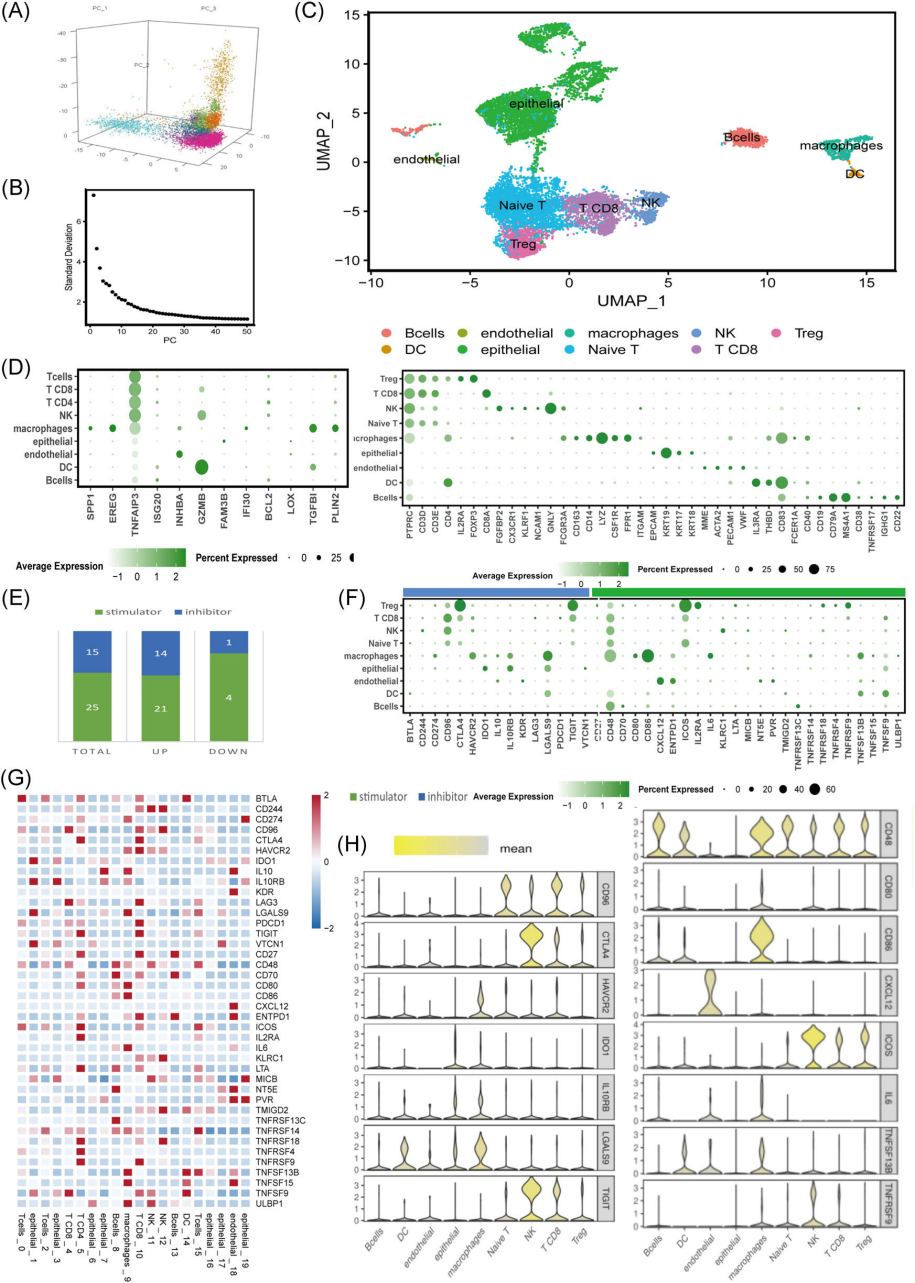
Single‐cell RNA sequencing profiling of the expression of immune checkpoint genes in the tumor microenvironment. (A) Three‐dimensional PCA plot of single‐cell sequence data in GSE171894. (B) t‐SNE plot showing the components of four patients' tissues. (C) Unsupervised clustering of viable cells from cervical squamous cell carcinoma tissues represented as an t‐SNE plot. (D) On left, dot plot of expression of immune‐related RNAs in nine clusters; on right, nine cell types. (E) The composition of 40 immune checkpoint genes (ICGs). (F) Dot plot showing the 40 ICGs in each cell type. (G) Boxplot showing the expression of 40 ICGs in 20 (0–19) clusters. (H) Violin plot of the most representative ICGs in nine cell types: left, inhibitor; right, stimulator. DC, dendritic cell; NK, natural killer; PCA, principal component analysis; t‐SNE, t‐distributed stochastic neighbor embedding

GSE171894 included four fresh samples obtained from patients with CSCC who underwent concurrent chemoradiotherapy. We had a total of 182,230 cells collected from the quality control process. We selected 30 principal components (Figure 6A,B) to subdivide these cells into 20 different clusters. These clusters were then divided into nine cell groups using the abovementioned cell markers (Figure 6C). The cell composition of these samples was primarily T‐cells and squamous epithelial tumor cells. The cell markers of the main cell lineages were visualized as a bubble chart according to the 20 clusters and 9 cell types (Figure 6D, right). A scatterplot was used to check the expression of an IRG signature in tumor tissues. The expression of *TNFAIP3* was higher in immune cells, especially in epithelial carcinoma, macrophages, and CD8^+^ T‐cells (Figure 6D, left), while *TGFBI*, *IFI30*, *SPP1*, and *EREG* were expressed more highly in macrophages than other cells.

We examined the expression of 70 common ICGs in TCGA–GTEx datasets. We tested the expression levels of 40 differentially expressed ICGs in the single‐cell sequencing data of GSE171884. The ratios of these 40 genes, including 36 upregulated genes and 4 downregulated genes, as well as 25 stimulators and 15 inhibitors, are presented in Figure 6E. The expressions of 40 ICGs in tumor tissues are visualized as a bubble chart according to 9 different cell types (Figure 6F,G). The average expression of ICGs can also be seen in Figure 9E (tumor vs. adjacent normal tissue). T‐cell exhaustion‐related ICIs (*CTLA4*, *CD96*, *HAVCR2*, and *TIGIT*) tended to be expressed in CD8^+^ T‐cells, natural killer (NK) cells, and Treg cells. *LGALS9*, *HAVCR2*, and *IL10RB* tended to be expressed in macrophages. The expressions of *CTLA4*, *TIGIT*, and *HAVCR2* in this depleted T‐cell subset were significantly higher than those of PD‐L1 (*CD274*) and *LAG3*, indicating that *TNFRSF9*, *CTLA4*, *TIGIT*, and *HAVCR2* could be better targeted for CC immunotherapy.

### 3.7 Single‐cell sequencing to defined the proposition of cells in tumor tissues and normal tissues

**Figure 7:**
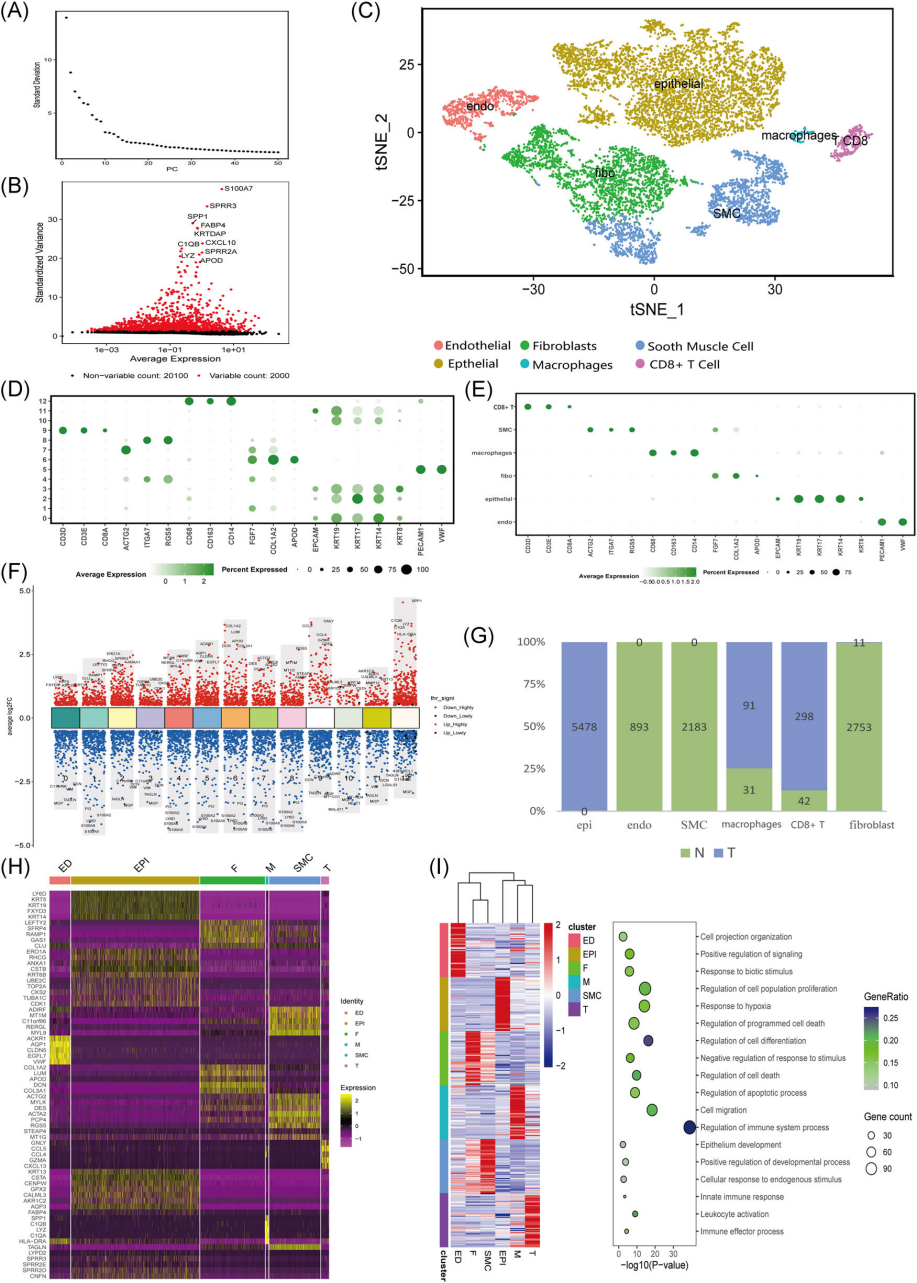
Single‐cell RNA sequencing profiling of the ecosystem in adjacent normal and cervical squamous cell carcinoma (CSCC) tumor tissues. (A) An elbow plot of 50 principal components for the clustering results. (B) Top 10 highly variable genes in GSE168652. (C) Unsupervised clustering of viable cells from CSCC and adjacent normal cervix tissues represented as a t‐SNE plot. (D) Dot plot showing the expression of cell markers in 13 clusters. (E) Dot plot showing the expression of cell markers in six cell types. (F) Dot plot showing the top variable genes in each cluster. (G) Histogram showing the proportion of each cell type in GSE168652: normal, green; tumor, blue. (H,I) Heatmap showing the expression of the specifically expressed gene in six cell types and the corresponding representative GO terms (on the right). GO, Gene Ontology; SMC, smooth muscle cell; t‐SNE, t‐distributed stochastic neighbor embedding

GSE168652 contained two samples, including adjacent normal tissues and tumor tissues, from the same patient with cervical squamous carcinoma. The raw data included 20,277 unique features and 11,394 cells from normal tissues and 13,104 cells from tumor cells. Through the quality control process, we determined 30 principal components used for clustering and t‐SNE with an elbow plot (Figure 7A). The top 10 highly variable genes (*S100A7*, *SPRR3*, *SPP1*, *FABP4*, *KRTDAP*, *APOD*, *SPRR2A*, *LYPD2*, *YZ*, and *C1QB*) are pictured in Figure 7B. Cells were first clustered into 13 clusters (Figure 7D), then finally defined as 6 different cell types (Figure 7C,E). Cervix tissue is composed of epithelial cells, smooth muscle cells, endothelial cells, and CD8^+^ T‐cells, and macrophages. The top five markers of the main cell lineages were visualized using a dot plot (Figure 7H). Most of the top variable genes in each group were IRGs, such as *SPP1*, which was most upregulated in macrophages, and *PI3*, which was downregulated in all epithelial cell groups (Figure 7F). The content of smooth muscle cells and stromal cells was decreased and the number of squamous epithelial cancer cells was increased greatly in cancer tissues. The contents of immune cells (CD8^+^ T‐cells and macrophages) in tumor tissues were higher than those in normal tissues (Figure 7G). The most specifically expressed gene's functional analysis (Figure 7I) of each cells type was conducted by Gene Ontology (GO) in online datasets Toppgene (https://toppgene.cchmc.org/enrichment.jsp). The GO terms showed “regulation of apoptotic process” and “cell migration” were enriched for macrophages. GO terms including “regulation of cell differentiation” and “regulation of cell death” were enriched for fibroblast cells. GO terms “regulation of hypoxia” and “regulation of programmed cells death” were enriched in epithelial cells (most of which were tumor cells). In smooth muscle cells, GO terms included “epithelium development” and “positive regulation of developmental process.” GO terms including “innate immune response” and “immune effector process” were enriched in T‐cells. GO terms “cell–cell signaling” and “positive regulation of signaling” were enriched in endo cells. The death of T‐cells was related to the prognosis of patients.

### 3.8 Analysis of CD8^+^ t‐cell transition states in normal and tumor samples

**Figure 8:**
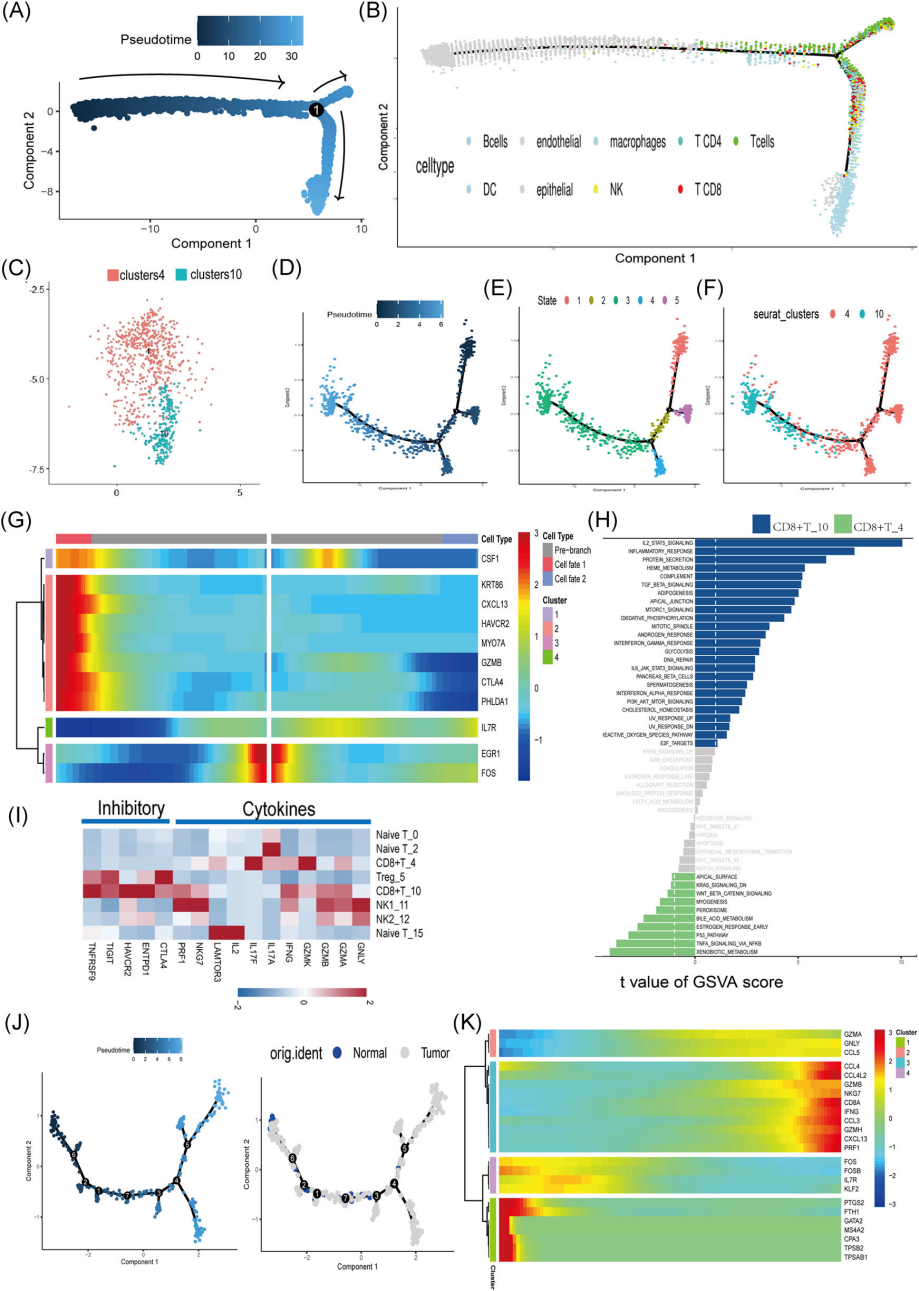
Pseudotime‐ordered analysis of CD8^+^ T‐cells. (A,B) Pseudotime‐ordered analysis of CD8^+^ T‐cells in GSE168652; different cell types are labeled by color. (C) t‐SNE plot showing the CD8^+^ T‐cells in GSE17184. (D–F) Pseudotime‐ordered analysis of CD8^+^ T cells; different stages and T‐cell subtypes (Clusters 4 and 10) are labeled by color. (G) Three‐dimensional heatmap showing the dynamic expression of genes along the pseudotime. (H) GSVA shows the pathway enrichment of Clusters 4 and 10. (I) Heatmap showing the expression of selected gene sets (inhibitory, cytokines) in T‐/natural killer subtypes. (J) Pseudotime–ordered analysis of CD8^+^ T‐cells in GSE17184. (K) Heatmap showing the dynamic expression of genes along the pseudotime. DC, dendritic cell; GSVA, gene set variation analysis; t‐SNE, t‐distributed stochastic neighbor embedding

We next utilized Monocle2 to perform a pseudotime analysis, exploring the cell transitions and dynamic immune states in all immune cells (Figure 8A,B) and CD8^+^ T‐cell subgroups (Figure 8D–F) by inferring the state trajectories in GSE171894. CD8^+^ T‐cells were divided into two clusters (Figure 8C, including Clusters 4 and 10), which expressed high levels of *GZMB* (GZMB^+^ CD8^+^ T‐cells) and *GZMK* (GZMK^+^ CD8^+^ T‐cells). *GZMB* is an apoptosis‐related gene, which cleaves and activates *GSDME* and caspase‐3, NK cells, and CD8^+^ killer lymphocytes, while chimeric antigen receptor T‐cells trigger pyrolysis. The trajectory of CD8^+^ T‐cells was visualized as a t‐SNE plot. GZMB^+^ CD8^+^ T‐cells were downstream in the development time, showing a state of decay. However, GZMK^+^ CD8^+^ T‐cells also tend to convert into GZMB^+^ CD8^+^ T‐cells. It was found that the expressions of *CTLA4* and *TIGIT* in GZMB^+^ CD8^+^ T‐cells were relatively higher than those in GZMK^+^ CD8^+^ T‐cells (Figure 8I). GZMB^+^ CD8^+^ T‐cells expressed high cytotoxic or exhausted signals (*CTLA‐4*, *TIGIT*, and *PDCD1*). According to reports, these molecules can inhibit the activity of CD8^+^ T‐cells by communicating with dendritic cells and macrophages. To observe the process of T‐cell exhaustion (time trajectory), we found through chronological analysis that CD8^+^ T‐cells from the active Cluster 2 tend to transform into debilitating T‐cells. In addition, clusters of T‐cells (Clusters 2, 4, and 5) representing exhaustion appeared at the end of the differentiation trajectory, during this process. *TIM‐3* and *TIGIT* have been reported as marker molecules of T‐cell exhaustion in chronic viral infections and cancer models. To investigate molecular interaction networks, we found that the high expression of the *Havcr2* gene in Cluster 9 may lead to immunosuppression, culminating in the failure of immunotherapy. Interestingly, we noticed that the toxicity gene and *CXCL13*, which dictates effective responses to programmed death ligand 1 blockade genes, are upregulated during tumor cell evolution (Figure 8G,H).

### 3.9 Macrophages play a vital role in cell–cell communication

**Figure 9:**
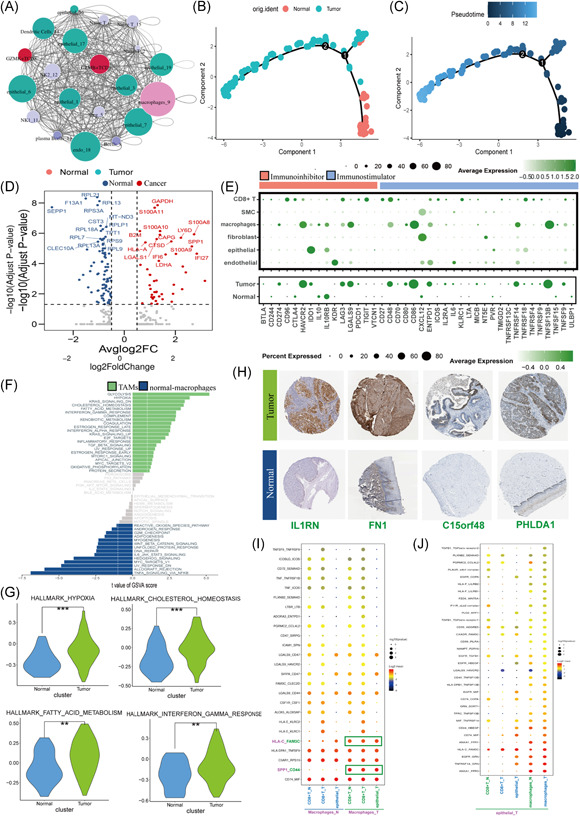
Analysis of macrophage transition states in tumor and adjacent normal samples. (A) The cell–cell communications network. (B,C) Pseudotime‐ordered analysis of macrophages from normal and tumor groups, which are labeled by color. (D) Volcano plot showing differentially expressed genes between macrophages in normal and tumor tissues; *p* < .05. (E) The average expressions of differentially expressed immune checkpoint genes: top, GSE171884; bottom, macrophages. (F) The GSVA of pathways enriched in macrophages in normal and tumor groups. (G) Violin plot showing the pathway enrichment of macrophages in normal and tumor groups. (H) Immunohistochemical map of four marker genes of SPP1^+^ tumor‐associated macrophages in normal and tumor tissues. (I,J) Bubble heatmap showing the mean attraction strength for selected ligand–receptor pairs between T‐cells, epithelial cells, and macrophages. GSVA, gene set variation analysis; SMC, smooth muscle cell; TAM, tumor‐associated macrophages

CellPhoneDB was used to calculate potential ligand–receptor pairs (GSE171884) and the cell–cell communication molecules in the TME. The interaction network was visualized by R. Macrophages possessed the most interaction pairs with cells from other lineages (Figure 9A), revealing the dominant role of macrophages in the TME. CD8^+^ T‐cells had the most connections with the macrophages, while macrophages had more connections with CD8^+^ T‐cells (especially GZMB^+^CD8^+^ T cells) and tumor epithelial cells. The macrophages in GSE168652 were divided into normal and tumor groups. Through a pseudotime analysis, we found that macrophages in adjacent normal tissues tended to convert into tumor‐infiltration macrophages (Figure 9B,C). The volcano plot shows that *SPP1* was highly expressed in tumor‐infiltration macrophages (Figure 9D). The immunohistochemical map of a marker of SPP1^+^ TAMs was also highly expressed in tumor tissues (Figure 9H). We calculated the average expression of ICGs in normal and tumor tissues and found that the average expression of ICGs in normal tissues was lower than that in tumor tissues (Figure 9E, upper). The average expression of ICGs in macrophages selected from normal tissues was lower than in tumor tissues (Figure 9E, lower). The gene set variation analysis results (Figure 9F) of normally arrived macrophages and TAMs indicate that TAMs are related to hypoxia, glycolysis, fatty metabolism, and inflammatory pathways. The top 27 ligand–receptors of macrophage immune cells are visualized in Figure 9I. SPP1‐CD44 plays a vital factor in cell–cell communications between macrophages and CD8^+^ T‐cells.

**Figure 10:**
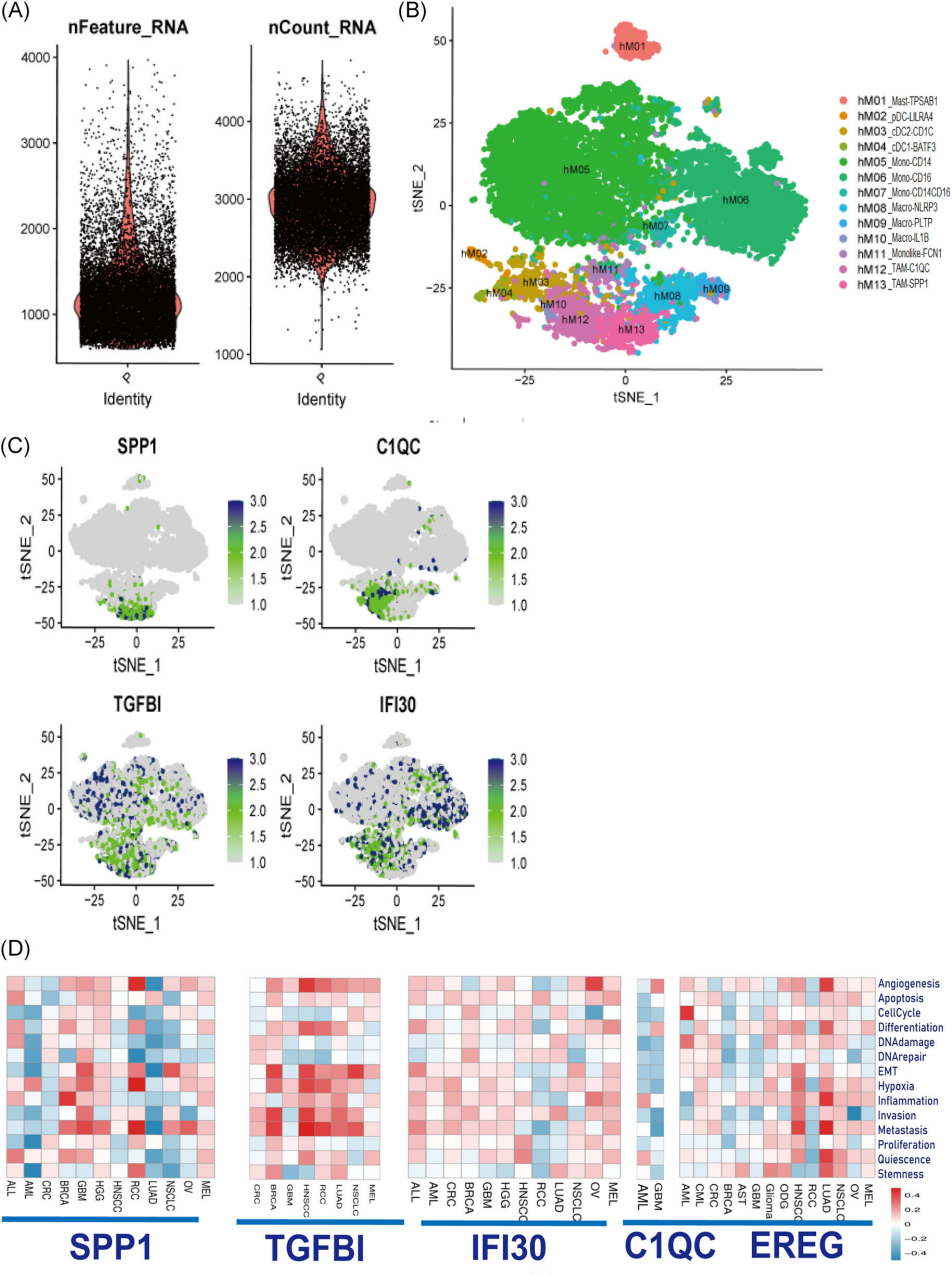
Single‐cell RNA sequencing profiling of the ecosystem in myeloid cell clusters. (A) Violin plots displaying the number of RNA features (nFeature_RNA) and absolute UMI counts (nCount_RNA). (B) Unsupervised clustering of myeloid cells represented as a t‐SNE plot. (C) Dimplot showing the expression of four genes (*C1QC*, *IFI30*, *SPP1*, and *TGFBI*). (D) The correlations between *C1QC*, *IFI30*, *SPP1*, *TGFBI*, and *EREG* and 14 different functional states. t‐SNE, t‐distributed stochastic neighbor embedding

### 3.10 Examining the expressions and functions of five genes in pan‐cancer analysis

The expressions of four genes (*SPP1*, *TGFBI*, *IFI30*, and *C1QC*) in myeloid cell clusters from colon cancer (GSE146771) are shown by a TSNE plot in Figure 10C, indicating that TGFBI^+^ M0‐like TAMs are a kind of CD14^+^ CD16^−^ blood‐derived monocytes. The expressions of five genes (*SPP1*, *TGFBI*, *IFI30*, *C1QC*, and *EREG*) are shown by a t‐SNE plot in Figure S2, indicating that TGFBI^+^ TAMs, SPP1^+^ TAMs, and CD16^+^ blood‐derived monocyte IFI30^+^ TAMs are three significantly different TAM subgroups in six cancers (AML, BRCA, GBM, HNSCC, LUAD, and MEL). The correlations between these genes and 14 functional states indicate that *TGFBI* has a strong positive correlation with EMT, hypoxia, metastasis, invasion, and angiogenesis. *SPP1* is found to be mainly involved in EMT, metastasis, and hypoxia.

## 4 DISCUSSION

Immunosuppression in the TME is the main obstacle to effective antitumor therapy for patients.^43^ Immune cells inhibit and kill tumor cells' antitumor immunity on the one hand, and promote tumor development and immune escape on the other hand. IRGs and expressed related proteins also play an important role in inhibiting or promoting tumor proliferation in the TME. Single‐cell genomes provide a viable strategy to understand heredity and phenotypic diversity at the single‐cell level, which may also help us to understand the complex ecosystem in tumors. The study showed that intratumor hypoxia was negatively correlated with tumor‐invasive T‐cell motility in a mouse fibrosarcoma model, especially at the core of solid tumors compared to their margins. TAMs constitute an important part of the plasticity and heterogeneity of TME,^44^ and the number of macrophages can account for up to 50% of some solid tumors.^44^

We tried to use Gaussian regression models to select the best logistic regression models to diagnose and predict the relapse of TCGA CC patients. Finally, we had a combination of six genes (*IFI30*, *SPP1*, *GZMB*, *EREG*, *ISG20*, and *FAM3B)*. We also examined the expression of HRGs, most of which had a strong correlation with IRGs. PPI network analysis showed that IRHs had strong correlations with each other, except *ISG20* (which was overlapped in IRG models). This study constructed risk score models for the IRHs. Patients in the low‐risk IRH group had a longer median OS than those in the high‐risk group, indicating that a hypoxic environment leads to a poor prognosis. The risk score showed that regulatory T‐cells, Th cells, and M0 and M1 macrophages were related to OS. We built a coexpression network between IRMs and IRLs. The IRLs associated with a good prognosis were AC092580.4, AC017002.1, and AC002331.1. The IRHs associated with a good prognosis were *ISG20* and *BCL2*. Finally, the IRHs associated with a bad prognosis were *LOX*, *PLIN2*, and *TGFBI*.

In comparing the occurrence and development of normal tissues and tumor tissues, we found that the expression levels of genes encoding activation and cytotoxic molecules, such as *GZMB*, *GZMH*, *GZMK*, *GZMA*, and *NKG7*, increased significantly and had strong correlations with signs of exhaustion. In terms of treatment, apart from traditional chemotherapy, targeted therapy, and antiangiogenesis therapy, novel immunotherapy based on ICGs has attracted increasing attention.^45^, ^46^, ^47^ We used a scatterplot to examine the expression of ICGs and found that it was expressed not only on T‐cells but also many immune cells, especially TAMs. Since the expressions of *TNFRSF9*, *CTLA4*, *TIGIT*, and *HAVCR2* in this depleted T‐cell subset were significantly higher than that of *CD274 (*PD‐L1), this indicates that *TNFRSF9*, *CTLA4*, *TIGIT*, and HAVCR2 may be better targets for immunotherapy, while *CXCL12* is upregulated during the evolution of CD8^+^ T‐cells and may be a marker of effective response to programmed death ligand 1 blockade. *GZMB*, *IFI30*, AC092580.4, AC017002.1, and AC002331.1 had strong correlations with ICGs (*CTLA4*, *TNFRSF9*, *CD86*, *TNFRSF13B*, *PDCD1*, and *CD48*), which are highly expressed in CD8^+^ T‐cells and TAMs.

In this study, we used single‐cell sequencing to identify the expression of IRRs in normal and tumor tissues. The expressions of SPP1, *IFI30*, and *TGFBI* in macrophages were higher than in other cells. *SPP1* and *TGFBI* were also poor prognostic markers and *IFI30* was a good prognostic marker of CC, respectively. The proportions of immune cells in tumor tissues appear higher than those in normal tissues. We checked the correlations between IRRs and macrophages, especially C1QC^+^ TAMs and SPP1^+^ TAMs. *IFI30* participates in the process of immune‐regulation, which plays a role in the promotion of C1QC^+^ TAMs to CD8^+^ T‐cells in the co‐expression network. The influence of C1QC^+^ TAMs and CD8^+^ T‐cells was not only affected by the interaction of ligands and receptors but also the regulation of the mRNA and lncRNA coexpression network. TGFBI^+^ M0‐like TAMs are a kind of blood‐derived monocyte‐derived TAMs gradually polarized to M2 under the influence of hypoxia‐influencing factors after entering tumor tissue. CD14^+^ TGFBI^+^ M0‐like TAMs compose the greatest proportion of total TAMs and are related to tumor angiogenesis, hypoxic necrosis, and tumor necrosis factor signaling and PI3K–AKT signaling pathways.

From the perspective of IRRs and immune‐related cells, we established immune‐related hypoxia prognosis, immune‐related lncRNA, and immune cell prognosis models. We also used Gaussian distribution to develop IRG models that have significant predictive properties for recurrence and diagnosis. We analyzed the interaction network of IRGs and lncRNA related to OS and analyzed the impact of hypoxia factors on the immune environment. Through two single‐cell sequencing analyses of CSCC, we identified the expression of these genes on tumors and normal cells. The GZMK^+^CD8^+^ T‐cells, GZMB^+^CD8^+^ T‐cells, IFI30^+^C1QC^+^ TAMs, and TAMs play vital roles in TME. We analyzed the communication relationship between tumor cells, then finally assessed the immune microenvironment formed by these IRRs and immune cells and the impact of these on the patient's recurrence, prognosis, and diagnosis. Immune system deficiency plays an important role in the progression of CC and the immune microenvironment of CC tissue is the key to personalized cancer treatment.

## AUTHOR CONTRIBUTIONS

Kun Deng contributed to the conception of the study. Ruiling Yin performed the data analyses and manuscript writing. Hongyan Han, Xuedong Tong, Yan Li, and Xiuming Zhai contributed significantly in writing the manuscript. All authors read and approved the final manuscript.

## CONFLICTS OF INTEREST

The authors declare no conflicts of interest.

## Supporting information

Supporting information.

Supporting information.

Supporting information.

## Data Availability

The raw datasets supporting the conclusions of this article are available via The Cancer Genome Atlas portal (https://portal.gdc.cancer.gov/projects/TCGA-CESC). GEO datases are available via GEO (https://www.ncbi.nlm.nih.gov/geo/query/acc.cgi?acc=GSE63514; https://www.ncbi.nlm.nih.gov/geo/query/acc.cgi?acc=GSE138080; https://www.ncbi.nlm.nih.gov/geo/query/acc.cgi?acc=GSE168652; https://www.ncbi.nlm.nih.gov/geo/query/acc.cgi?acc=GSE171894). The single‐cell RNA sequencing data generated in this paper is available in GSE16852, GSE171894, and GSE146771 datasets. GEO datasets including GSE138080, GSE63514 datasets. TCGA‐CESC datasets from UCSC XENA (http://xena.ucsc.edu/) were also used in this study. All remaining relevant data are available in the article, or from the corresponding author.
