# Supplementary figures and images for: Characterizing the landscape of cervical squamous cell carcinoma immune microenvironment by integrating the single‐cell transcriptomics and RNA‐Seq
Source: Immun Inflamm Dis. 2022 May 11;10(6):10.1002/iid3.608. doi: 10.1002/iid3.608 (PMC9091987; doi:10.1002/iid3.608)

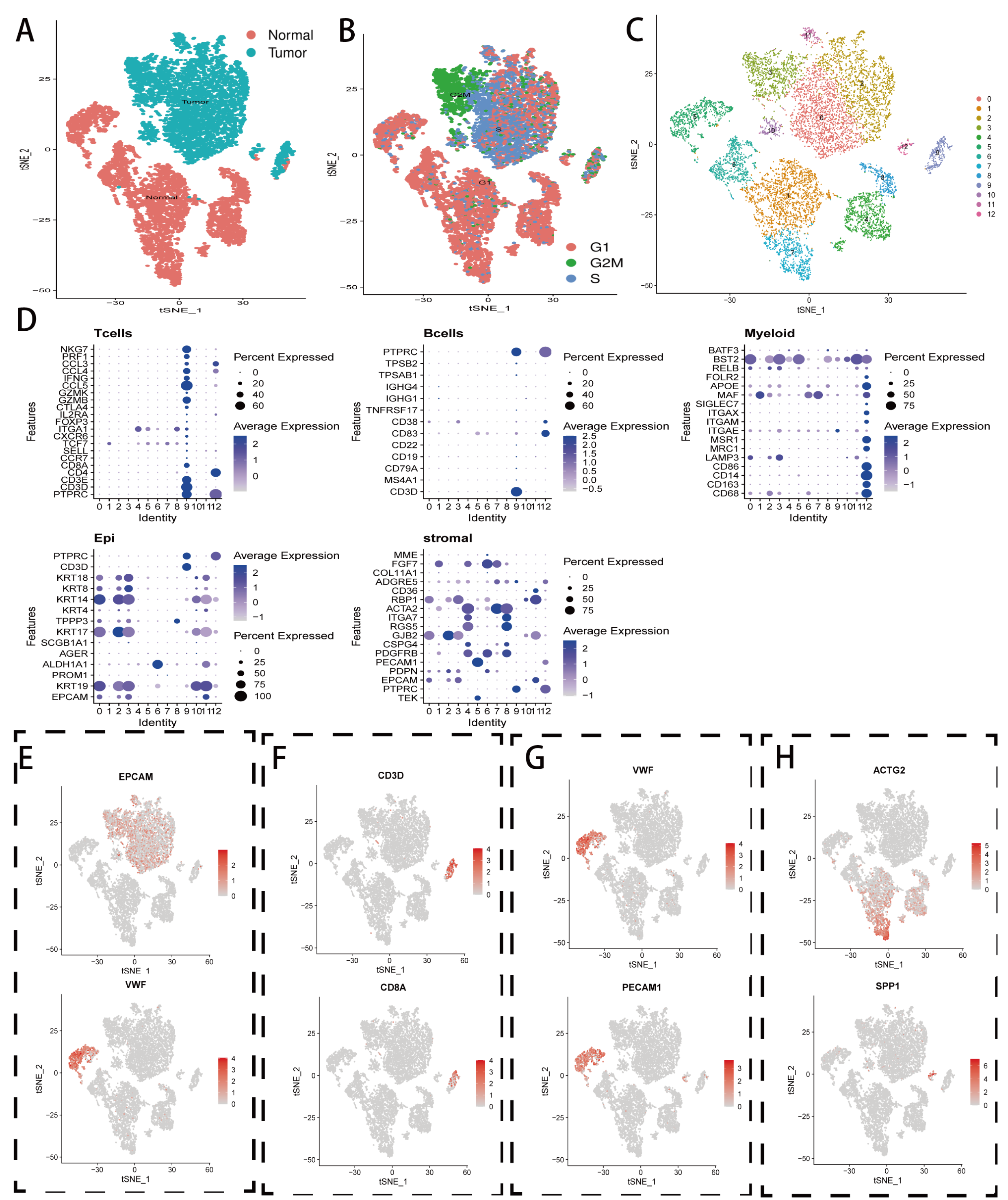

Supplement: Supplementary file 1 — Supporting information. [file IID3-10--s003.tif]

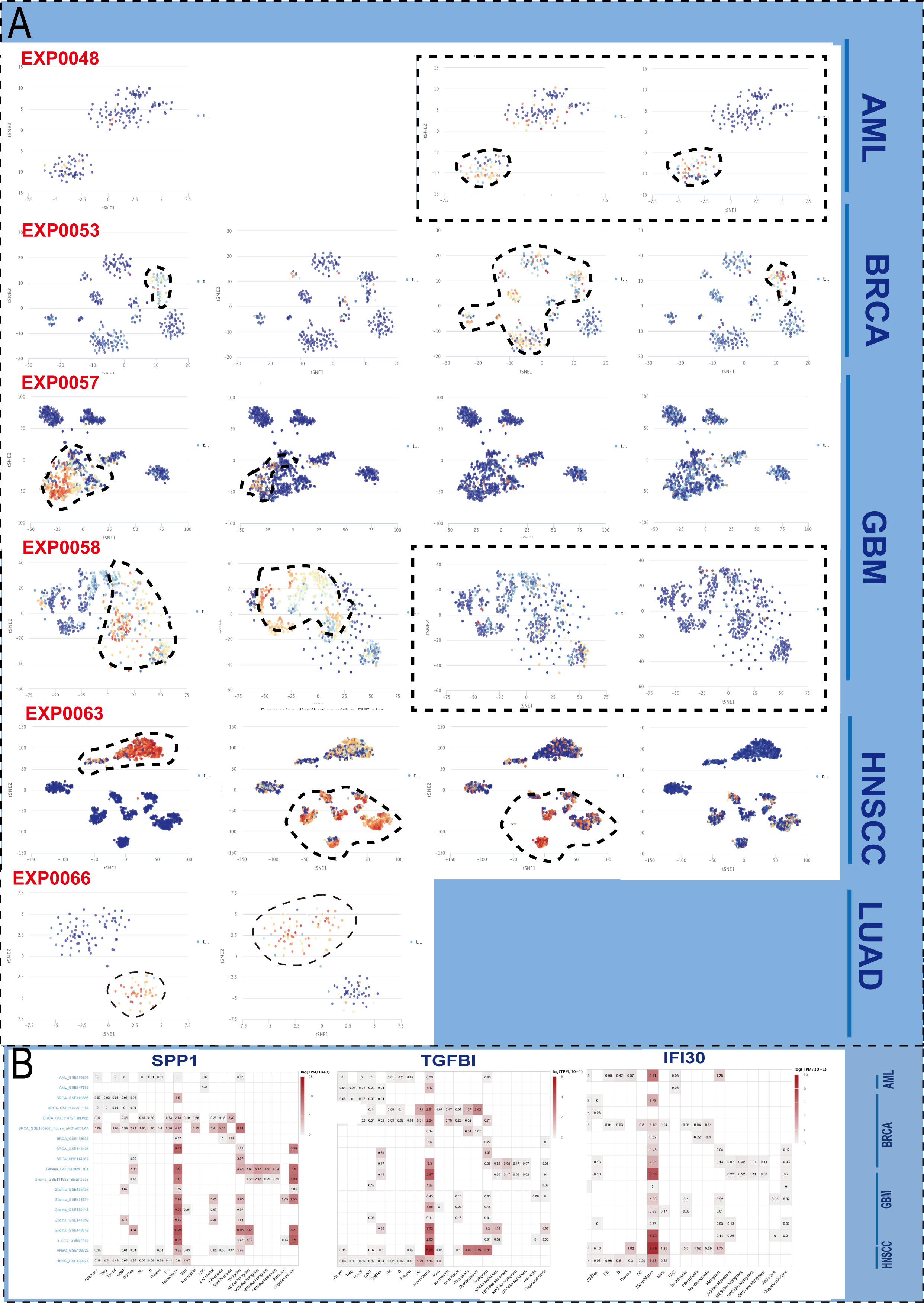

Supplement: Supplementary file 2 — Supporting information. [file IID3-10--s002.tif]
