# Supplementary material for: Characterizing the landscape of cervical squamous cell carcinoma immune microenvironment by integrating the single‐cell transcriptomics and RNA‐Seq
Source: Immun Inflamm Dis. 2022 May 11;10(6):10.1002/iid3.608. doi: 10.1002/iid3.608 (PMC9091987; doi:10.1002/iid3.608)
Supplement: Supplementary file 3 — Supporting information. [file IID3-10--s001.docx]

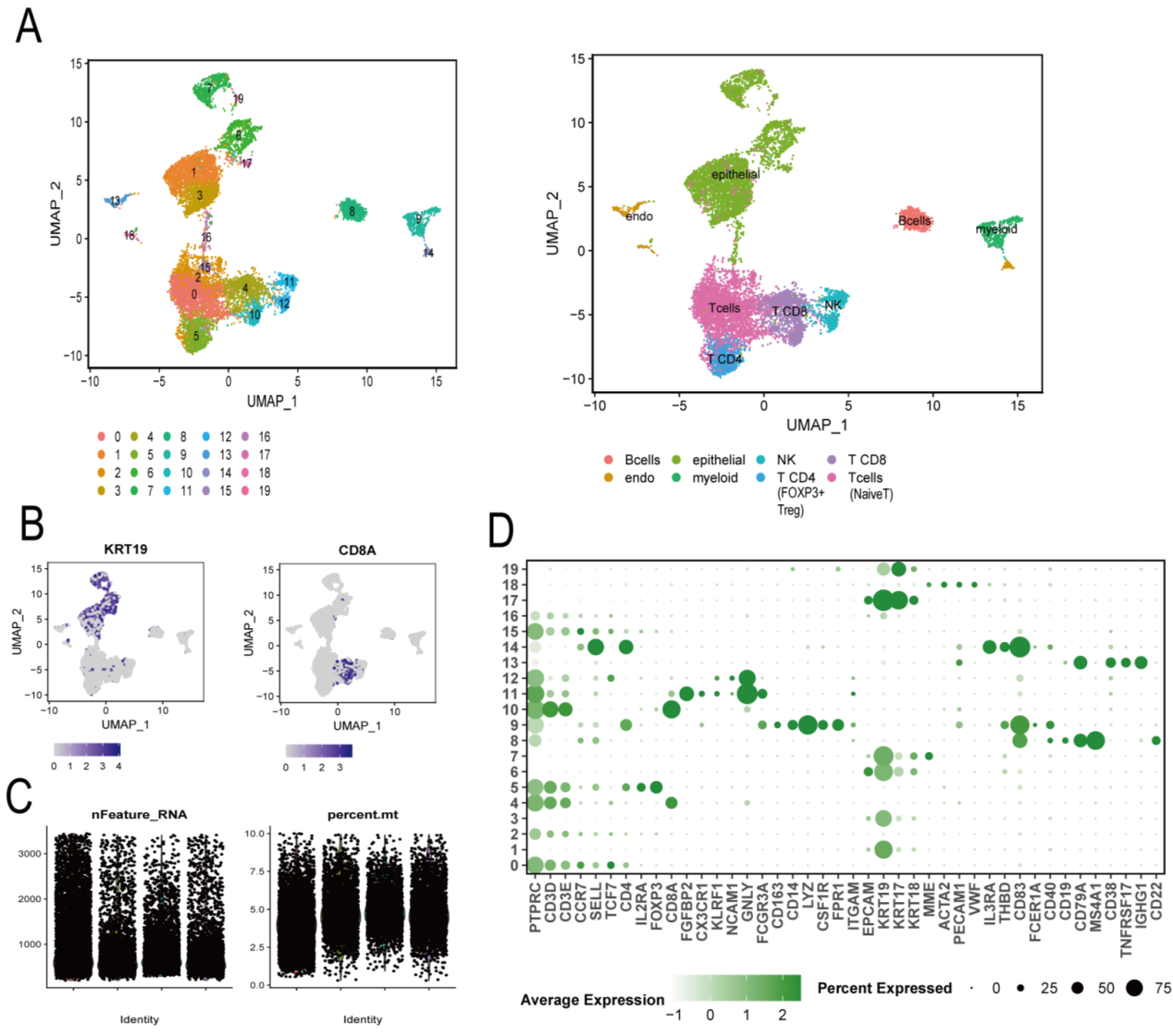


**Figure S3:** Quality control of GSE171894. (**A)** Unsupervised clustering of cells from CSCC tissues, represented as a UMAP plot. Left: clusters; Right: celltypes. **(B)** Dimplot plots showing expression levels of marker mRNA in distinct clusters cells. **(C)** Violin plots displaying the number of RNA features (nFeature_RNA) and mitochondrial gene expression (percent.mt). **(D)** Dotplot plots showing the expression levels of marker mRNA in distinct clusters cells.
